# 2,3-Dibromo-1-(4-chloro­phen­yl)-3-(5-nitro-2-fur­yl)propan-1-one

**DOI:** 10.1107/S1600536810046829

**Published:** 2010-11-20

**Authors:** Hoong-Kun Fun, Chin Sing Yeap, Shobhitha Shetty, Balakrishna Kalluraya

**Affiliations:** a X-ray Crystallography Unit, School of Physics, Universiti Sains Malaysia, 11800 USM, Penang, Malaysia; b Department of Studies in Chemistry, Mangalore University, Mangalagangotri, Mangalore 574 199, India

## Abstract

In the title compound, C_13_H_8_Br_2_ClNO_4_, the linking –CHBr–CHBr– fragment is disordered over two orientations with refined site occupancies of 0.512 (11) and 0.488 (11). The dihedral angle between the furan ring and the phenyl ring is 21.86 (16)°. In the crystal, the mol­ecules are linked into [011] chains by inter­molecular C—H⋯O hydrogen bonds.

## Related literature

For applications of nitro­furan derivatives, see: Holla *et al.* (1986 ▶, 1987 ▶, 1992 ▶). For the synthesis, see: Rai *et al.* (2008 ▶). For stability of the temperature controller used in the data collection, see: Cosier & Glazer (1986 ▶).
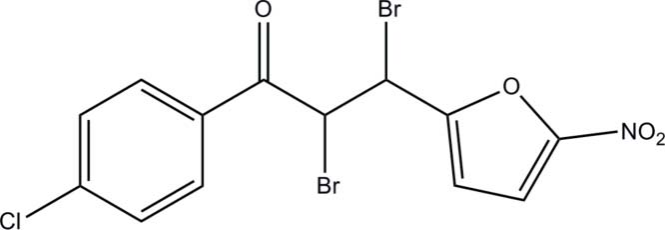


## Experimental

### 

#### Crystal data

C_13_H_8_Br_2_ClNO_4_
*M* *_r_* = 437.47
Triclinic, 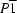

*a* = 8.4932 (11) Å
*b* = 9.4501 (12) Å
*c* = 10.5665 (14) Å
α = 92.731 (2)°
β = 107.000 (2)°
γ = 114.299 (2)°
*V* = 725.32 (16) Å^3^
*Z* = 2
Mo *K*α radiation
μ = 5.79 mm^−1^
*T* = 100 K
0.28 × 0.18 × 0.10 mm

#### Data collection

Bruker APEXII DUO CCD diffractometer
Absorption correction: multi-scan (*SADABS*; Bruker, 2009 ▶) *T*_min_ = 0.297, *T*_max_ = 0.587
13696 measured reflections
3856 independent reflections
3398 reflections with *I* > 2σ(*I*)
*R*_int_ = 0.032

#### Refinement

*R*[*F* ^2^ > 2σ(*F* ^2^)] = 0.028
*wR*(*F* ^2^) = 0.064
*S* = 1.20
3856 reflections
227 parameters
H-atom parameters constrained
Δρ_max_ = 0.36 e Å^−3^
Δρ_min_ = −0.41 e Å^−3^

### 

Data collection: *APEX2* (Bruker, 2009 ▶); cell refinement: *SAINT* (Bruker, 2009 ▶); data reduction: *SAINT*; program(s) used to solve structure: *SHELXTL* (Sheldrick, 2008 ▶); program(s) used to refine structure: *SHELXTL*; molecular graphics: *SHELXTL*; software used to prepare material for publication: *SHELXTL* and *PLATON* (Spek, 2009 ▶).

## Supplementary Material

Crystal structure: contains datablocks global, I. DOI: 10.1107/S1600536810046829/hb5734sup1.cif

Structure factors: contains datablocks I. DOI: 10.1107/S1600536810046829/hb5734Isup2.hkl

Additional supplementary materials:  crystallographic information; 3D view; checkCIF report

## Figures and Tables

**Table 1:** Hydrogen-bond geometry (Å, °)

*D*—H⋯*A*	*D*—H	H⋯*A*	*D*⋯*A*	*D*—H⋯*A*
C9*A*—H9*AA*⋯O1^i^	0.98	2.26	3.199 (6)	160
C4—H4*A*⋯O3^ii^	0.93	2.46	3.184 (4)	135

Symmetry codes: (i) 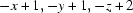; (ii) 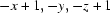.

**Fig. 1.**
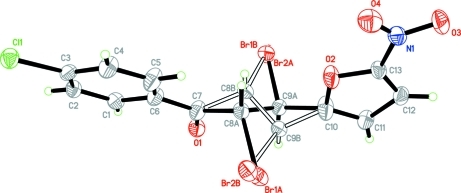
The molecular structure of the title compound with 50% probability ellipsoids for non-H atoms. Open bonds indicate the minor component.

**Fig. 2.**
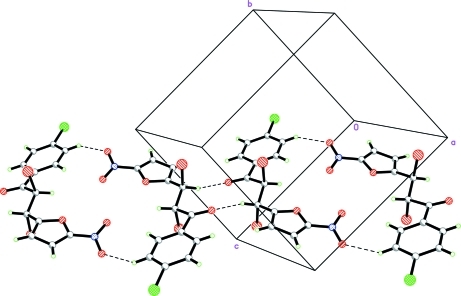
The crystal packing of title compound, showing the molecules are linked into a chain approximately along [011]. Intermolecular hydrogen bonds are shown as dashed lines. Only major disorder component is shown.

## supplementary crystallographic information

### Comment

Nitrofurans are class of synthetic compounds characterized by the presence of 5-nitro-2-furyl group. The presence of nitro group in position-5 of the molecule conferred antibacterial activity (Holla *et al.*, 1986). A large number of nitrofurans have attained commercial utility as antibacterial agents in humans and in veterinary medicine because of their broad spectrum of activity (Holla *et al.*, 1987, 1992). The dibromopropanones were obtained by the bromination of 1-aryl-3-(5-nitro-2-furyl)-2-propen-1-ones. Acid-catalysed condensation of acetophenones with nitrofural diacetate in acetic acid yielded the required 1-aryl-3-(5-nitro-2-furyl)-2-propen-1-ones called chalcones (Rai *et al.*, 2008).

The dibromopropanone of the title compound is disordered over two positions with refined site occupancies of 0.512 (11) and 0.488 (11) (Fig. 1). The furan ring and the phenyl ring make dihedral angle of 21.86 (16)°. In the crystal structure, the molecules are linked into chains approximately along the [0 1 1] by intermolecular C9A—H9AA···O1 and C4—H4A···O3 hydrogen bonds (Fig. 2, Table 1).

### Experimental

1-(4-Chlorophenyl)-3-(5-nitro-2-furyl)-2-propen-1-one (0.01 mol) was dissolved in glacial acetic acid (25 ml) by gentle warming. A solution of bromine in glacial acetic acid (30% *w*/*v*) was added to it with constant stirring till the yellow color of the bromine persisted. The reaction mixture was kept aside at room temperature for overnight. Crystals of dibromopropanones which separated out were collected by filtration and washed with ethanol and dried. It was then recrystallized from glacial acetic acid. Colourless blocks were obtained from 1:2 mixtures of DMF and ethanol by slow evaporation.

### Refinement

All hydrogen atoms were positioned geometrically [C–H = 0.93 or 0.98 Å] and refined using a riding model [*U*_iso_(H) = 1.2*U*_eq_].

### Crystal data

**Table:**

C_13_H_8_Br_2_ClNO_4_	*Z* = 2
*M**_r_* = 437.47	*F*(000) = 424
Triclinic, *P*1	*D*_x_ = 2.003 Mg m^−^^3^
Hall symbol: -P 1	Mo *K*α radiation, λ = 0.71073 Å
*a* = 8.4932 (11) Å	Cell parameters from 6344 reflections
*b* = 9.4501 (12) Å	θ = 2.8–30.1°
*c* = 10.5665 (14) Å	µ = 5.79 mm^−^^1^
α = 92.731 (2)°	*T* = 100 K
β = 107.000 (2)°	Block, colourless
γ = 114.299 (2)°	0.28 × 0.18 × 0.10 mm
*V* = 725.32 (16) Å^3^	

### Data collection

**Table:**

Bruker APEXII DUO CCD diffractometer	3856 independent reflections
Radiation source: fine-focus sealed tube	3398 reflections with *I* > 2σ(*I*)
graphite	*R*_int_ = 0.032
φ and ω scans	θ_max_ = 29.0°, θ_min_ = 2.4°
Absorption correction: multi-scan (*SADABS*; Bruker, 2009)	*h* = −11→11
*T*_min_ = 0.297, *T*_max_ = 0.587	*k* = −12→12
13696 measured reflections	*l* = −14→14

### Refinement

**Table:**

Refinement on *F*^2^	Primary atom site location: structure-invariant direct methods
Least-squares matrix: full	Secondary atom site location: difference Fourier map
*R*[*F*^2^ > 2σ(*F*^2^)] = 0.028	Hydrogen site location: inferred from neighbouring sites
*wR*(*F*^2^) = 0.064	H-atom parameters constrained
*S* = 1.20	*w* = 1/[σ^2^(*F*_o_^2^) + (0.*P*)^2^ + 0.7632*P*] where *P* = (*F*_o_^2^ + 2*F*_c_^2^)/3
3856 reflections	(Δ/σ)_max_ = 0.002
227 parameters	Δρ_max_ = 0.36 e Å^−^^3^
0 restraints	Δρ_min_ = −0.41 e Å^−^^3^

### Special details

**Table:**

Experimental. The crystal was placed in the cold stream of an Oxford Cryosystems Cobra open-flow nitrogen cryostat (Cosier & Glazer, 1986) operating at 100.0 (1) K.
Geometry. All e.s.d.'s (except the e.s.d. in the dihedral angle between two l.s. planes) are estimated using the full covariance matrix. The cell e.s.d.'s are taken into account individually in the estimation of e.s.d.'s in distances, angles and torsion angles; correlations between e.s.d.'s in cell parameters are only used when they are defined by crystal symmetry. An approximate (isotropic) treatment of cell e.s.d.'s is used for estimating e.s.d.'s involving l.s. planes.
Refinement. Refinement of *F*^2^ against ALL reflections. The weighted *R*-factor *wR* and goodness of fit *S* are based on *F*^2^, conventional *R*-factors *R* are based on *F*, with *F* set to zero for negative *F*^2^. The threshold expression of *F*^2^ > σ(*F*^2^) is used only for calculating *R*-factors(gt) *etc*. and is not relevant to the choice of reflections for refinement. *R*-factors based on *F*^2^ are statistically about twice as large as those based on *F*, and *R*- factors based on ALL data will be even larger.

### Fractional atomic coordinates and isotropic or equivalent isotropic displacement parameters (Å^2^)

**Table:**

	*x*	*y*	*z*	*U*_iso_*/*U*_eq_	Occ. (<1)
Br1A	0.6289 (7)	0.5168 (5)	0.7250 (5)	0.0356 (5)	0.512 (11)
Br2A	0.3194 (8)	0.1148 (6)	0.9250 (8)	0.0268 (7)	0.512 (11)
Br1B	0.3164 (8)	0.1124 (6)	0.9164 (8)	0.0282 (9)	0.488 (11)
Br2B	0.5885 (6)	0.5025 (6)	0.7004 (5)	0.0321 (5)	0.488 (11)
Cl1	−0.35654 (9)	0.23719 (8)	0.24802 (6)	0.02977 (14)	
O1	0.2656 (3)	0.4225 (2)	0.86289 (18)	0.0295 (4)	
O2	0.6347 (3)	0.1530 (2)	0.76863 (18)	0.0345 (5)	
O3	0.8567 (3)	−0.0787 (3)	0.7618 (2)	0.0470 (6)	
O4	0.6542 (3)	−0.0476 (3)	0.5983 (2)	0.0393 (5)	
N1	0.7574 (3)	−0.0148 (3)	0.7148 (2)	0.0300 (5)	
C1	−0.0395 (3)	0.3544 (3)	0.6339 (2)	0.0249 (5)	
H1A	−0.0353	0.4008	0.7151	0.030*	
C2	−0.1827 (3)	0.3272 (3)	0.5175 (2)	0.0245 (5)	
H2A	−0.2771	0.3520	0.5201	0.029*	
C3	−0.1840 (3)	0.2625 (3)	0.3966 (2)	0.0239 (5)	
C4	−0.0475 (4)	0.2207 (3)	0.3908 (3)	0.0331 (6)	
H4A	−0.0507	0.1768	0.3089	0.040*	
C5	0.0930 (4)	0.2451 (4)	0.5083 (3)	0.0370 (7)	
H5A	0.1844	0.2161	0.5056	0.044*	
C6	0.0998 (3)	0.3129 (3)	0.6312 (3)	0.0282 (5)	
C7	0.2512 (4)	0.3473 (3)	0.7606 (3)	0.0350 (6)	
C8A	0.4124 (8)	0.3173 (8)	0.7467 (6)	0.0243 (15)	0.512 (11)
H8AA	0.3686	0.2261	0.6753	0.029*	0.512 (11)
C8B	0.3687 (8)	0.2509 (8)	0.7864 (6)	0.0206 (14)	0.488 (11)
H8BA	0.3450	0.1875	0.7008	0.025*	0.488 (11)
C9A	0.5118 (8)	0.2949 (8)	0.8801 (5)	0.0231 (16)	0.512 (11)
H9AA	0.5574	0.3905	0.9473	0.028*	0.512 (11)
C9B	0.5707 (8)	0.3651 (8)	0.8459 (6)	0.0217 (15)	0.488 (11)
H9BA	0.5934	0.4294	0.9306	0.026*	0.488 (11)
C10	0.6760 (4)	0.2677 (4)	0.8747 (3)	0.0405 (7)	
C11	0.8308 (4)	0.2911 (3)	0.9747 (3)	0.0291 (5)	
H11A	0.8863	0.3628	1.0561	0.035*	
C12	0.8923 (3)	0.1848 (3)	0.9317 (3)	0.0276 (5)	
H12A	0.9958	0.1724	0.9780	0.033*	
C13	0.7676 (3)	0.1059 (3)	0.8086 (3)	0.0255 (5)	

### Atomic displacement parameters (Å^2^)

**Table:**

	*U*^11^	*U*^22^	*U*^33^	*U*^12^	*U*^13^	*U*^23^
Br1A	0.0434 (14)	0.0312 (8)	0.0500 (15)	0.0229 (10)	0.0299 (11)	0.0179 (9)
Br2A	0.0255 (13)	0.0341 (15)	0.0279 (8)	0.0156 (11)	0.0148 (8)	0.0104 (8)
Br1B	0.0261 (13)	0.0214 (13)	0.0353 (17)	0.0091 (9)	0.0099 (8)	0.0066 (8)
Br2B	0.0354 (11)	0.0366 (8)	0.0392 (11)	0.0216 (9)	0.0234 (8)	0.0171 (7)
Cl1	0.0302 (3)	0.0323 (3)	0.0260 (3)	0.0153 (3)	0.0070 (2)	0.0031 (2)
O1	0.0310 (9)	0.0303 (10)	0.0275 (9)	0.0170 (8)	0.0066 (8)	−0.0019 (7)
O2	0.0341 (10)	0.0431 (11)	0.0270 (9)	0.0272 (9)	−0.0004 (8)	−0.0104 (8)
O3	0.0382 (12)	0.0446 (13)	0.0647 (15)	0.0293 (11)	0.0128 (11)	−0.0026 (11)
O4	0.0475 (12)	0.0398 (12)	0.0333 (11)	0.0217 (10)	0.0156 (10)	−0.0021 (9)
N1	0.0277 (11)	0.0272 (11)	0.0409 (13)	0.0138 (9)	0.0180 (10)	0.0027 (10)
C1	0.0273 (12)	0.0260 (12)	0.0254 (12)	0.0142 (10)	0.0118 (10)	0.0017 (10)
C2	0.0235 (12)	0.0278 (13)	0.0265 (12)	0.0141 (10)	0.0105 (10)	0.0043 (10)
C3	0.0196 (11)	0.0223 (12)	0.0260 (12)	0.0073 (9)	0.0062 (9)	0.0012 (9)
C4	0.0289 (13)	0.0387 (15)	0.0301 (13)	0.0174 (12)	0.0067 (11)	−0.0082 (11)
C5	0.0260 (13)	0.0458 (17)	0.0363 (14)	0.0202 (13)	0.0041 (11)	−0.0143 (13)
C6	0.0231 (12)	0.0288 (13)	0.0298 (13)	0.0128 (10)	0.0052 (10)	−0.0066 (10)
C7	0.0273 (13)	0.0362 (15)	0.0372 (14)	0.0185 (12)	0.0017 (11)	−0.0118 (12)
C8A	0.026 (3)	0.025 (3)	0.023 (3)	0.014 (2)	0.008 (2)	0.001 (2)
C8B	0.028 (3)	0.020 (3)	0.017 (2)	0.012 (2)	0.009 (2)	0.003 (2)
C9A	0.022 (3)	0.025 (3)	0.023 (2)	0.012 (2)	0.007 (2)	−0.001 (2)
C9B	0.024 (3)	0.023 (3)	0.025 (3)	0.012 (2)	0.015 (2)	0.007 (2)
C10	0.0381 (16)	0.0500 (18)	0.0316 (14)	0.0307 (15)	−0.0030 (12)	−0.0162 (13)
C11	0.0253 (12)	0.0356 (14)	0.0253 (12)	0.0147 (11)	0.0065 (10)	−0.0004 (10)
C12	0.0227 (12)	0.0318 (14)	0.0314 (13)	0.0133 (11)	0.0114 (10)	0.0083 (11)
C13	0.0248 (12)	0.0267 (12)	0.0323 (13)	0.0153 (10)	0.0141 (10)	0.0064 (10)

### Geometric parameters (Å, °)

**Table:**

Br1A—C8A	2.102 (8)	C5—C6	1.395 (3)
Br2A—C9A	2.008 (9)	C5—H5A	0.9300
Br1B—C8B	1.962 (11)	C6—C7	1.488 (4)
Br2B—C9B	2.059 (8)	C7—C8A	1.553 (5)
Cl1—C3	1.732 (3)	C7—C8B	1.586 (6)
O1—C7	1.211 (3)	C8A—C9A	1.492 (9)
O2—C13	1.343 (3)	C8A—H8AA	0.9800
O2—C10	1.377 (3)	C8B—C9B	1.511 (9)
O3—N1	1.235 (3)	C8B—H8BA	0.9800
O4—N1	1.220 (3)	C9A—C10	1.534 (5)
N1—C13	1.433 (3)	C9A—H9AA	0.9800
C1—C2	1.377 (3)	C9B—C10	1.511 (6)
C1—C6	1.398 (3)	C9B—H9BA	0.9800
C1—H1A	0.9300	C10—C11	1.349 (4)
C2—C3	1.385 (3)	C11—C12	1.420 (3)
C2—H2A	0.9300	C11—H11A	0.9300
C3—C4	1.385 (3)	C12—C13	1.349 (4)
C4—C5	1.378 (4)	C12—H12A	0.9300
C4—H4A	0.9300		
			
C13—O2—C10	104.5 (2)	C9B—C8B—C7	109.4 (5)
O4—N1—O3	125.4 (2)	C9B—C8B—Br1B	106.2 (5)
O4—N1—C13	119.5 (2)	C7—C8B—Br1B	112.8 (4)
O3—N1—C13	115.2 (2)	C9B—C8B—H8BA	109.5
C2—C1—C6	120.7 (2)	C7—C8B—H8BA	109.5
C2—C1—H1A	119.6	Br1B—C8B—H8BA	109.5
C6—C1—H1A	119.6	C8A—C9A—C10	110.9 (5)
C1—C2—C3	119.0 (2)	C8A—C9A—Br2A	105.0 (5)
C1—C2—H2A	120.5	C10—C9A—Br2A	114.6 (4)
C3—C2—H2A	120.5	C8A—C9A—H9AA	108.7
C2—C3—C4	121.5 (2)	C10—C9A—H9AA	108.7
C2—C3—Cl1	119.61 (18)	Br2A—C9A—H9AA	108.7
C4—C3—Cl1	118.85 (19)	C10—C9B—C8B	107.3 (5)
C5—C4—C3	119.0 (2)	C10—C9B—Br2B	122.0 (4)
C5—C4—H4A	120.5	C8B—C9B—Br2B	99.1 (4)
C3—C4—H4A	120.5	C10—C9B—H9BA	109.2
C4—C5—C6	120.7 (2)	C8B—C9B—H9BA	109.2
C4—C5—H5A	119.7	Br2B—C9B—H9BA	109.2
C6—C5—H5A	119.7	C11—C10—O2	110.9 (2)
C5—C6—C1	119.0 (2)	C11—C10—C9B	131.8 (3)
C5—C6—C7	123.3 (2)	O2—C10—C9B	115.6 (3)
C1—C6—C7	117.7 (2)	C11—C10—C9A	130.1 (3)
O1—C7—C6	122.1 (2)	O2—C10—C9A	114.4 (3)
O1—C7—C8A	121.7 (3)	C9B—C10—C9A	31.3 (2)
C6—C7—C8A	114.6 (3)	C10—C11—C12	106.6 (2)
O1—C7—C8B	113.5 (3)	C10—C11—H11A	126.7
C6—C7—C8B	122.3 (3)	C12—C11—H11A	126.7
C8A—C7—C8B	29.3 (2)	C13—C12—C11	104.8 (2)
C9A—C8A—C7	108.0 (5)	C13—C12—H12A	127.6
C9A—C8A—Br1A	99.1 (4)	C11—C12—H12A	127.6
C7—C8A—Br1A	114.4 (4)	O2—C13—C12	113.2 (2)
C9A—C8A—H8AA	111.6	O2—C13—N1	116.0 (2)
C7—C8A—H8AA	111.6	C12—C13—N1	130.8 (2)
Br1A—C8A—H8AA	111.6		
			
C6—C1—C2—C3	−2.0 (4)	C7—C8B—C9B—C10	177.3 (3)
C1—C2—C3—C4	1.8 (4)	Br1B—C8B—C9B—C10	55.3 (5)
C1—C2—C3—Cl1	−176.6 (2)	C7—C8B—C9B—Br2B	−54.9 (5)
C2—C3—C4—C5	−0.4 (4)	Br1B—C8B—C9B—Br2B	−176.9 (3)
Cl1—C3—C4—C5	178.1 (2)	C13—O2—C10—C11	1.0 (4)
C3—C4—C5—C6	−0.9 (5)	C13—O2—C10—C9B	168.0 (4)
C4—C5—C6—C1	0.7 (5)	C13—O2—C10—C9A	−157.4 (4)
C4—C5—C6—C7	−177.8 (3)	C8B—C9B—C10—C11	−144.5 (5)
C2—C1—C6—C5	0.8 (4)	Br2B—C9B—C10—C11	102.5 (5)
C2—C1—C6—C7	179.4 (3)	C8B—C9B—C10—O2	51.9 (6)
C5—C6—C7—O1	171.3 (3)	Br2B—C9B—C10—O2	−61.1 (6)
C1—C6—C7—O1	−7.3 (5)	C8B—C9B—C10—C9A	−43.5 (6)
C5—C6—C7—C8A	5.8 (5)	Br2B—C9B—C10—C9A	−156.5 (8)
C1—C6—C7—C8A	−172.8 (4)	C8A—C9A—C10—C11	156.7 (5)
C5—C6—C7—C8B	−26.5 (6)	Br2A—C9A—C10—C11	−84.6 (5)
C1—C6—C7—C8B	154.9 (4)	C8A—C9A—C10—O2	−50.1 (7)
O1—C7—C8A—C9A	36.2 (7)	Br2A—C9A—C10—O2	68.6 (5)
C6—C7—C8A—C9A	−158.2 (4)	C8A—C9A—C10—C9B	49.6 (6)
C8B—C7—C8A—C9A	−45.6 (6)	Br2A—C9A—C10—C9B	168.2 (7)
O1—C7—C8A—Br1A	−73.0 (6)	O2—C10—C11—C12	−0.5 (4)
C6—C7—C8A—Br1A	92.5 (4)	C9B—C10—C11—C12	−164.7 (5)
C8B—C7—C8A—Br1A	−154.8 (7)	C9A—C10—C11—C12	153.5 (5)
O1—C7—C8B—C9B	−63.8 (6)	C10—C11—C12—C13	−0.2 (3)
C6—C7—C8B—C9B	132.7 (4)	C10—O2—C13—C12	−1.1 (3)
C8A—C7—C8B—C9B	49.6 (6)	C10—O2—C13—N1	−179.8 (3)
O1—C7—C8B—Br1B	54.2 (5)	C11—C12—C13—O2	0.9 (3)
C6—C7—C8B—Br1B	−109.4 (4)	C11—C12—C13—N1	179.3 (3)
C8A—C7—C8B—Br1B	167.5 (7)	O4—N1—C13—O2	13.2 (4)
C7—C8A—C9A—C10	−178.9 (4)	O3—N1—C13—O2	−166.3 (2)
Br1A—C8A—C9A—C10	−59.4 (5)	O4—N1—C13—C12	−165.2 (3)
C7—C8A—C9A—Br2A	56.7 (5)	O3—N1—C13—C12	15.3 (4)
Br1A—C8A—C9A—Br2A	176.2 (3)		

### Hydrogen-bond geometry (Å, °)

**Table:**

*D*—H···*A*	*D*—H	H···*A*	*D*···*A*	*D*—H···*A*
C9A—H9AA···O1^i^	0.98	2.26	3.199 (6)	160
C4—H4A···O3^ii^	0.93	2.46	3.184 (4)	135

Symmetry codes: (i) −*x*+1, −*y*+1, −*z*+2; (ii) −*x*+1, −*y*, −*z*+1.
